# In the face of war: examining sexual vulnerabilities of Acholi adolescent girls living in displacement camps in conflict-affected Northern Uganda

**DOI:** 10.1186/1472-698X-12-38

**Published:** 2012-12-28

**Authors:** Sheetal H Patel, Herbert Muyinda, Nelson K Sewankambo, Geoffrey Oyat, Stella Atim, Patricia M Spittal

**Affiliations:** 1 Centre for Health Evaluation and Outcome Sciences, Vancouver, BC, Canada; 2 School of Population and Public Health, University of British Columbia, Vancouver, BC, Canada; 3 Child Health and Development Centre, Makerere University, Kampala, Uganda; 4 Makerere University College of Health Sciences, Kampala, Uganda; 5 Child Protection Programme, Save the Children Liberia, Monrovia, Liberia; 6 Community-based Researcher, Gulu Town, Uganda

**Keywords:** Adolescent girls, Conflict, Sexual vulnerability, Displacement camps, Northern Uganda, Acholi, Qualitative, HIV/AIDS

## Abstract

**Background:**

Adolescent girls are an overlooked group within conflict-affected populations and their sexual health needs are often neglected. Girls are disproportionately at risk of HIV and other STIs in times of conflict, however the lack of recognition of their unique sexual health needs has resulted in a dearth of distinctive HIV protection and prevention responses. Departing from the recognition of a paucity of literature on the distinct vulnerabilities of girls in time of conflict, this study sought to deepen the knowledge base on this issue by qualitatively exploring the sexual vulnerabilities of adolescent girls surviving abduction and displacement in Northern Uganda.

**Methods:**

Over a ten-month period between 2004–2005, at the height of the Lord’s Resistance Army insurgency in Northern Uganda, 116 in-depth interviews and 16 focus group discussions were held with adolescent girls and adult women living in three displacement camps in Gulu district, Northern Uganda. The data was transcribed and key themes and common issues were identified. Once all data was coded the ethnographic software programme ATLAS was used to compare and contrast themes and categories generated in the in-depth interviews and focus group discussions.

**Results:**

Our results demonstrated the erosion of traditional Acholi mentoring and belief systems that had previously served to protect adolescent girls’ sexuality. This disintegration combined with: the collapse of livelihoods; being left in camps unsupervised and idle during the day; commuting within camp perimeters at night away from the family hut to sleep in more central locations due to privacy and insecurity issues, and; inadequate access to appropriate sexual health information and services, all contribute to adolescent girls’ heightened sexual vulnerability and subsequent enhanced risk for HIV/AIDS in times of conflict.

**Conclusions:**

Conflict prevention planners, resettlement programme developers, and policy-makers need to recognize adolescent girls affected by armed conflict as having distinctive needs, which require distinctive responses. More adaptive and sustainable gender-sensitive reproductive health strategies and HIV prevention initiatives for displaced adolescent girls in conflict settings must be developed.

## Background

The intersection between war, displacement and HIV/AIDS is particularly striking in Northern Uganda. The over two-decade war between the Government of Uganda (GoU) and Joseph Kony and his rebel force, the Lord’s Resistance Army (LRA), has been characterized by the killing and maiming of civilians, child abductions, widespread displacement, various forms of sexual and gender-based violence, and the destruction of the social and economic fabric of society [1-3]. These experiences have left the entire population traumatized and at a heightened risk of contracting HIV/AIDS.

Northern Uganda, or Acholiland as it is often referred to, is comprised of three districts, Gulu, Kitgum, and Pader and spans an area of 28,000 square kilometres, equivalent to the size of Rwanda. Over a million and a half people reside between the three districts, of which 99% are of Acholi ethnicity and 1% other tribes. Acholiland is a vast and sparsely populated area with considerable agriculture and livestock development potential. The Acholi are traditionally a people of agriculturalists and livestock breeders and prior to the conflict, ninety percent of people in the northern region lived in rural areas.

In 1986, after President Museveni took power in Uganda, the rebel factions that have mobilized to resist this government can be characterized by transitions of increased terror, with Joseph Kony being the craft perpetrator of some of the greatest human rights violations the world has ever seen. Even though Joseph Kony and his rebel force, the LRA, professed to fight a spiritual war for the Acholi people against the GoU and its military, the Ugandan People’s Defence Forces (UPDF), the majority of Acholi people did not respect or voluntarily assist the LRA. This repudiation can largely be attributed to the enhanced phase of terror inflicted upon the civilian population after the peace talks brokered by Betty Bigombe in 1994 fell apart. Rape, landmines and the mass abduction of children as combatants became the signature work of the LRA. To this day, the facial mutilations of women who had their lips, ears and noses severed at gunpoint are visible in displacement camp settings.

When the peace talks failed, the Sudanese government allegedly began to heavily support Joseph Kony [4]. By providing safe refuge in the form of encampments, land to cultivate, materials to build homesteads, hospitals for treatment of war-related injury and even pharmaceuticals for treatment of common infections such as sexually transmitted infections (STIs), the Sudanese supported the LRA by enabling them to systemize their incursions into Uganda from protected base camps in the Sudan. To a large extent the Sudanese support of the LRA, including weapons, ammunition and landmines, was the key factor in consolidating Joseph Kony’s reign of terror as the longest child hostage crisis in human history [4].

By October of 1996 the casualty levels were high, the numbers of abducted children numbered close to five thousand and, the conflict had intensified with rebel incursions becoming a normal part of daily life. Due to the focused efforts of the LRA the GoU facilitated the shift of villagers into Internally Displaced Peoples (IDP) camps, and approximately 210,000 villagers moved from their homes into government-sanctioned camps [4]. Voluntary movement was not considered an option. At the time, most of the camps were located in the Kilak, Aswa and Nwoya counties of Gulu district, as they were the most affected by rebel incursion. By the year 2000, there were approximately 23 government-recognized camps in the region [4].

In the wake of September 11^th^, 2001, and with increased pressure by the US government on Islamic states supporting terrorism, the Sudanese and Ugandan governments committed to improving bilateral relations. In March 2002, the Ugandan government launched ‘Operation Iron Fist’ (OIF), a military offensive against the LRA. Thousands of ground troops and air support were deployed. The government’s intention was to resolve the situation in the North using military force, and diminish the effects of what was becoming an international embarrassment for the government. In response, the LRA rebels poured back across the Ugandan border and sought revenge against the civilian population with intensified attacks on communities, increasing abductions and forced recruitment. The number of abducted children under 18 years of age jumped from approximately 12,000 as of June 2002, to nearly double that by June 2003 and at least 30,000 by May 2004 [5].

As LRA attacks and abductions increased, and districts in Northern Uganda previously relatively unaffected by the war became targets of the LRA’s insurgency, unprecedented numbers of people fled their homes and were displaced into IDP camps all over Acholiland. The total number of people displaced and aid-dependent swelled. While in August 2001 there were an estimated 480,000 IDPs, by 2005 the total number of displaced persons had expanded to over 1.8 million, which accounted for over 90 percent of the population of Northern Uganda [6-8]. At that time, nearly 70 percent of the displaced population was under 25 years of age [9]. With the majority of people in the northern region now in camps, an unintended consequence of OIF was the complete destruction of Northern Uganda’s economic base, agriculture. Like many conflict-affected regions across Africa, Acholiland - once a very fertile region of the country - was left neglected, untended and uncultivated.

### Women/girls, HIV vulnerability, and conflict

In times of conflict, adolescent girls are caught in a web of vulnerability created by the social disarray of war. Indeed, the power imbalances that heighten girls’ sexual vulnerability and enhance their disproportionate risk for HIV/AIDS become even more pronounced during conflict and displacement [6,10]. Contributing factors which increase the spread of HIV in the context of conflict include: breakdown of family and other social and community structures; increased dependence on men for physical or economic security; lack of access to sexual health information, health care and social services; increased sexual and gender-based violence, and; sexual interaction between civilians and combatants [6,11,12]. All of these factors represent critical challenges for the international community with regard to programme response in times of conflict and, more often than not, resources available fall short of the scope and complexity of the challenges [13].

Even though Uganda has won international recognition for its progress in HIV research and prevention, regional disparities persist. According to the Ugandan Sero-Behavioural Survey last conducted in 2004/05, HIV prevalence among young people aged 15–29 was estimated at 9 percent among females and 7.1 percent among males for the whole North Central Region, while the national average figures were 8 percent and 5 percent respectively [14]. Overall prevalence in Northern Uganda was 8.2%, the highest in the country. However, these estimates are limited, because HIV prevalence rates are reported regionally and this makes it difficult to ascertain and compare rates in the districts most impacted by conflict, and therefore limits opportunity for further analysis by sub-group.

Although it is recognized that the relationship between conflict and HIV transmission is highly complex and contextualized and, that the ultimate effects of conflict (i.e. augmenting or reducing HIV risk) are continuously debated, [6,8,11,15-17] it has been well established that in conflict settings adolescent girls experience heightened sexual vulnerability and increased exposure opportunity to HIV [1,11,17-19]. Mock et al. ( [2004]) state that vulnerability factors (i.e. poverty, malnutrition, lack of health services), and exposure opportunity factors (i.e. increased sexual violence and physical violence) are the basic determinants of population-level HIV risk and that, seen in this way, conflict should be viewed as a key determinant of HIV vulnerability [11].

A study conducted by the Women’s Commission for Refugee Women and Children demonstrated that among children living in IDP camps, the idea of a ‘safe passage’ from childhood to adolescence to adulthood no longer existed and that this is particularly the case for adolescent mothers [20]. Furthermore, research suggests that even though adolescent girls are gradually receiving more attention from programme developers and policy-makers, the child protection community, academia and other stakeholders must strive to deepen the knowledge base on adolescent girls’ distinctive vulnerabilities to HIV/AIDS during times of war in order to inform more effective advocacy and programme interventions [3,11,13]. The main objective of this study was to provide a better understanding of adolescent girls’ enhanced risk for HIV infection in conflict settings by examining the sexual vulnerabilities of Acholi adolescent girls living in displacement camps in Northern Uganda, to inform the development of appropriate sexual education and HIV prevention initiatives in this population group.

## Methods

### Study design and sampling strategy

To understand the distinct vulnerabilities of conflict-affected Acholi adolescent girls and to in-turn develop culturally sensitive HIV/AIDS programming, the perspective of risk and HIV vulnerability of adolescent girls, before and during conflict, must be thoroughly understood in the context of Acholi culture. As such, a qualitative approach including interviews with a broad sample of girls and women who were resident in one of three IDP camps in Gulu district (Pabbo, Palenga or, Awer) was deemed necessary to fulfill our research objective. Between November 2004-September 2005, participants were recruited using convenience sampling, on the basis of proximity, age and consent. This sampling strategy enabled researchers to select informants who were easily accessible, either by nature of their geographic proximity or their connection to other research participants. The study team collaborated with hired community members in each site, who assisted with the recruitment process by mobilizing prospective age-eligible participants.

#### Sample size

Over a ten-month period, 116 in-depth interviews and 16 focus group discussions (including 6–10 participants each) were held in the three camps with adolescent girls and adult women. Disaggregated, these consisted of 59 in-depth interviews and 8 focus group discussions with adolescent girls aged 14–19 years and, 57 in-depth interviews and 8 focus group discussions with adult women over the age of 30 years. A breakdown of the sample by IDP camp is presented in Table 1.

**Table 1:** Breakdown of sample based on IDP camp

	**Pabbo**	**Palenga**	**Awer**	**Total**
**Indepths (*n*** **= 116)**				
Adolescent girls	30	15	14	59
Adult women	27	14	16	57
**Focus Groups (*n*** **= 16)**				
Adolescent girls	3	3	2	8
Adult women	3	3	2	8

### Data collection

#### Informed consent

All research participants provided written or thumbprint informed consent. The permission of the head of household was obtained before any members of the family participated in the study. In addition, every adolescent under the age of 18 must have had the consent of their parents/guardian before they could participant in the study. A number of adolescent girls qualified as mature minors (i.e. girls under the age of 18 who were married, currently pregnant, or already having children) and, therefore, were permitted to give their own consent. The consent forms were discussed in the local language of Luo to ensure consistency and clarity in the consent process. Only after full understanding of study procedures had been established, written or thumbprint informed consent was obtained for participation in the study. It was made clear to all involved that they could refuse to answer any question and that their withdrawal from the research at any point would warrant no negative consequences.

#### Interviews and focus group discussions

Highly trained, female, Acholi interviewers, bilingual in the local language of Luo and English, conducted the in-depth interviews and facilitated the focus group discussions. The interviews and group discussions explored issues including: camp/bush living; knowledge and perception of risk for HIV and other STIs; traditional ways of communicating/learning about sex and sexuality, and; perception of current availability and/or barriers to sex-related information and HIV care services. The in-depth one-on-one interviews took a narrative approach, using a series of prompts to allow participants to relate their understandings and perceptions of risk for HIV/AIDS, their knowledge of traditional ways of learning about sexuality and, the availability and barriers to sex-related information in the context of war and displacement camp living. New concerns, vague answers and contradictory ideas from the in-depth interviews were formulated into discussion topics and became part of the topic list used to guide the focus group discussions. The group discussions provided an opportunity for participants to compare their stories with others and, through interaction, articulate particular meanings of HIV-related vulnerabilities and the impact of the on-going conflict for themselves. Group interviews proved useful, as many experiences and perceptions were only identified when participants could compare them with the experiences and perceptions of other participants.

To ensure confidentiality of the data collected, all participants were identified by unique, pre-printed numeric study ID numbers (no names or personal identifiers were collected). In addition, all interviews and focus groups were conducted in total privacy by highly trained, same-sex interviewers, and no information was disclosed to respondents’ family members. All interviews took place at the participant’s home in a private and quiet place of their choosing and focus groups discussions were conducted in spare meeting rooms at local organization’s offices, in complete confidentiality. As discussion of sensitive issues and traumatic experiences could precipitate feelings of distress, every study participant was offered immediate referrals for psychosocial support as well as general healthcare; all referrals were made at the participant’s request.

### Ethical considerations

Ethical approval was obtained from the Institutional Review Boards from the University of British Columbia, Vancouver, Canada and Makerere University, Kampala, Uganda.

### Data analysis

With participants’ permission, all interviews and group discussions were audiotaped and subsequently transcribed by the Acholi research team in Uganda. A thematic analysis of the data was conducted using procedures for qualitatively derived data [21-23]. The main aim of the analysis was to identify issues that commonly emerge in the interviews and focus groups, which help to explain the sexual vulnerability of displaced adolescent girls. After the data collection process was complete in two of three of the IDP camps (Pabbo and Palenga), members of the research team familiarized themselves with the data by repeatedly reading interview and group discussion transcripts while at the same time making notes on identified patterns in the data. The notes were then collated and sorted into categories and sub-categories representing themes/recurring issues that were common across many different interviews and group discussions. Dynamic dialogue and reflection with our Acholi colleagues regarding the emerging thematic trends informed the development of our analytic framework. An initial coding scheme was developed that the team together agreed was a fair representation of the emerging thematic trends in vulnerability reflected in the narratives and stories of the adolescent girls and women we interviewed from Pabbo and Palenga IDP camps. Subsequent interviews conducted in Awer camp were used to confirm or refute the themes/trends in vulnerability that had previously been identified in the other two camps. As more data were collected and analyzed, coding categories were refined. Credibility of the thematic analysis was continually evaluated with the members of our research team, leaders and experts in HIV/AIDS and, community members. Community feedbacks were essential in developing a deeper understanding of patterns of risk and vulnerability and finding agreement among camp membership related to the main themes identified in the narratives and experiences of participants. All decisions related to analysis were carefully recorded. Once all the data had been collected and coded, we used the ethnographic computer software package, ATLAS, to organize, compare and contrast key themes and categories generated in the in-depth interviews and focus group discussions. Excerpts from the narrative data presented in this manuscript are annotated with type of interviewee [adolescent girl(s) or adult woman/women], prefixed by (I) for one-on-one interviews or (G) for group discussions.

## Results

Three broad thematic areas emerged that described the sexual vulnerabilities of displaced adolescent girls: 1) Understanding the Devastation of the Present: Erosion of Cultural Processes and Belief Systems and the Collapse of Livelihood; 2) Living in ‘Double Jeopardy’: Vulnerability in the Light of Day and Vulnerability at Night, and; 3) Access to Reproductive and Sexual Health Information.

### THEME I: Understanding the devastation of the present: erosion of cultural processes and belief systems and the collapse of livelihood

Through early discussions with our Acholi research team and the analysis of the data, it became clear that traditionally and in the not too distant past, Acholi girls experienced significantly high levels of personal and sexual security prior to marriage. The structured mentoring roles played by female relatives combined with belief systems that mitigated against premenstrual sex and emphasized the culturally imbued concept of personal and community consent, represented a child protection system that placed a high value on the Acholi girl’s personal and familial dignity and served to protect her sexuality.

#### The role of the grandmother (‘adaa’) and aunt (‘wayo’)

In-depth interviews confirmed findings from existing literature on traditional Acholi mentoring systems. Specifically, grandmothers and the huts in which they resided were pivotal in the transmission of sexual health knowledge and mentoring of young girls. While girls were indeed raised by their mothers and fathers, the grandmother (‘adaa’) played a significant role in the upbringing of female children in offering mentoring and guidance via story telling in the privacy of her own hut [24,25]. The grandmothers generally taught the girls about men, the importance of not moving in with men before they menstruated, marriage, the clan system and incest taboos. As one girl who went through the mentoring process describes:

“We girls had a separate sleeping hut. My grandmother would come to our sleeping hut in the night. She told us how to behave and be respectful to other people. She used to tell us that a responsible girl does not answer back when she is talked to, and when she is being addressed, she sits down. If she is called she comes and kneels down, and listens to what she is told…that is what a bright girl does.” [(I) Adolescent Girl]

Similar to other Ugandan cultures, among the Acholi, intergenerational taboos exist regarding discussing sex. Mothers did not speak to their daughter’s about sex, and this role became associated with the ‘*wayo’*. The ‘*wayo*’, the father’s sister, was responsible for socialization of young girls into (proper) womanhood and assumed a sister-like role in the girl’s life. She taught her nieces about various aspects of maturing in Acholi culture, relayed sexual health information, and was expected to be a trusted secret keeper, a mentor, and confidant. As one woman describes:

“A wayo is a mentor, secret keeper at the onset of menstruation…to teach you everything … even how to have sex with your husband, because you may have grown up with ignorance. She may teach you that if you have a man and he has called you to go to his home … when you are to have sex you are to do this and that, otherwise you may not be enticing enough for him.” [(I) Adult Woman]

#### Premenstrual sex and the idea of consensual sex in Acholi culture

Traditionally the age of sexual debut for Acholi girls was between the ages of 16 and 18 years and could only occur after the female child had had a number of regular menstrual cycles [24]. According to interviews with adult women, prior to the war premenstrual sex was forbidden and was the cause of much consternation and stigma for both families involved. In fact, if a child was discovered having sex prior to menstruation the incident became a kin and community issue and was dealt with in the same manner as a sexual assault that took place in the bush. Rituals would have to be involved that included compensation from the man’s family. As one woman explains:

“An appeasement ritual is performed at the spot where the rape took place. This would involve a goat (provided by the boys family) being slaughtered at the exact spot of the rape.” [(I) Adult Woman]

This ritual was justified, as it was believed that premenstrual sex was the cause of future problems with fertility for the girl who had been violated. Seen in this way, a female child’s sexual debut was culturally sanctioned and became a family/kinship issue.

When a girl had menstruated, she could begin receiving suitors. The men would first arrive at the family compound where aunties, mothers and grandmothers would determine a relationship’s potential by eliminating the possibility that the couple could be in some way related. After it was determined that a union was possible, courting could commence and eventually the suitor obtained permission to propose. However, prior to bride wealth negotiations a girl had to actually provide her *consent* to the relationship and to sexual relations. One woman differentiated between traditional Acholi customs related to courting and the current state of affairs in the camps:

“If a boy began to court you he had to first present himself at your home and your mother would have to see him … when you consented to a boy’s proposal it was done by giving him such articles like beads, handkerchiefs, bangles … but the girls of today they consent with their bodies … by the time you get to know your son’s fiancée he will have had relationships with many girls … In the past a boy couldn’t just come and use a girl.” [(G) Adult Women]

Parents expressed grief that adolescents would now rarely comply with them, and that they no longer observed traditional moral values including: respecting elders; protecting their virginity; abstaining from pre-marital sex and pregnancies, and; recognizing the importance of marriage. Adding to the change in behavioural norms was the powerlessness that girls and women in our study described as being associated with the congestion of IDP camp living. Displacement camp living represented to both girls and women a shift in their sexual decision-making from *control and consent* to passivity and acquiescence.

#### Collapse of livelihood

Operation Iron Fist, the military offensive against the LRA launched by the Uganda government in 2002, created circumstances where more people were displaced from traditional homesteads and interrupted significantly the households ability to generate income from the sale of harvests including groundnuts, sim-sim and maize. When families cannot dig and become completely food-aid dependant, the most vulnerable of all are the daughters. The majority of participants (76% of individual interviews) lamented the lack of meaningful agricultural production since the commencement of displacement camp living and commented that with the exception of performing casual labour for people who had access to their land, there were very few employment opportunities especially for girls and women, in or outside the camps. This lack of dynamism in IDP camp economies has kept displaced families in perpetual poverty and has led to fundamental changes in the way women and men lead their lives and provide for their families. As one participant suggests:

“Life is hard because there is hardly any means to earn income. It is because we do not have access to land due to insecurity. The land we dig, we rent or borrow but it is so little. Since there is no useful cultivation, we cannot afford to pay fees for our children, feed them and look after ourselves.” [(G) Adult Women]

Mothers reported that this inability to provide for their families left women and girls powerless and economically dependent on men, particularly adolescent girls.

“Every time you go to get water, a girl will wash herself and go to chat or stand by peoples kiosks … eventually the owner or trader in the kiosk will begin to seduce her. He will first buy for her pens, soap, books and other small presents … he will eventually ask for sex … he will say I have helped you a lot so you should also help me …they have to give him what he desires.” [(G) Adult Women]

Furthermore, the inability of families to meet the subsistence needs of their daughters was directly related to the decision of many girls to participate in ‘survival sex’, even exchanging sex for menstrual pads and biscuits. One 13-year old girl describes her experience:

“The only alternative is for you to go to a boy/man, so that he can help you with money to cater for things like clothing, food and other necessities. If you spend a night with the army officer at the barracks, the next day you will change to another man, provided he gives you some money.” [(I) Adolescent Girl]

The pain and indignity that mothers felt of being so powerless in the face of such predation was clear. Most camp residents survived on food that was supplied by the World Food Programme (WFP), and women also relied on these rations to earn some income. Mothers commented that they would either sell portions of their food rations to buy the things that their daughters felt they needed, or give rations to their daughters to sell themselves to try and prevent sex-for-exchange relationships.

“We sell part of it to buy soap, clothes and other necessities…now we are being taught that no matter how much food ration you get, you must give a little to the girls so at least she can get some money to buy herself a petticoat (skirt slip) or a panty … girls must come to us for the things they need.” [(G) Adult Women]

### THEME II: Living in double jeopardy: vulnerability in the light of Day and vulnerability at night

#### Vulnerability in the light of day

Before the on-set of war an Acholi girl who was not in school would accompany her mother to the garden, weed and clean the garden, be involved in the harvest and storage of foodstuffs, collect firewood, and haul water. This served as part of the socialization process and offered protection, as a child would be under the guidance and control of an adult. Since the war however, as many families were so afraid of abduction of their young ones, particularly girls around the ages of 10, 11 and 12 years, they started leaving their daughters behind within the relative protection of the camps.

“In the past while we were in our homesteads a boy or girl could have her own garden…but now because of the camp and insecurity, which limits movement to the gardens, the girls have become lazy.” [(G) Adult Women]

“Ever since the government ordered everybody into the camp, due to the escalation of rebel activity, I haven’t allowed my children to move far away from the camp. It’s I who volunteers and exposes myself to carry firewood, cassava and anything with which to feed them, so they could remain in the camp. They shouldn’t go anywhere; they should remain in the camps to avoid abduction.” [(I) Adult Woman]

It was commonly reported (64% of individual interviews with adult women) that in the absence of adult supervision girls who were left behind in the camps during the day were vulnerable to potential harms, including sexual predation. As one woman observes:

“Supposing you leave for the garden, she will also leave for an unknown destination. You return from the garden only to hear rumours about her bad conduct all over the camp. In our absence she will have gone to have sex with a boy in the daytime. In my case, my daughter is spending time with boys in their huts.” [(G) Adult Women]

This situation whereby mothers were forced to leave girls idle and unsupervised in the camps during the day due to security concerns was quite different from leaving your daughter at home alone in a village-setting, prior to the war, where homes were at least three kilometres apart. For a child to move from household to household looking for where to sleep and to get something to eat was totally unacceptable in the Acholi culture. It was disgusting, worrying, and it undermined the socialization process. Parents reported feeling incompetent and useless in such circumstances and felt that their power and rights to protect their families and children had been taken away with the on-set of displacement camp living.

Contributing to this vulnerable situation was large numbers of adolescent girls dropping out of school and/or diminished school attendance. A common situation described in focus group discussions with mothers in the camps was adolescent girls leaving school due to early pregnancy and early marriage or because they could not afford the uniform and other costs associated with schooling (i.e. books, pencils), despite the fact that primary school is free in Uganda. Furthermore, some children missed classes because they were afraid of being abducted on the way to and from school while others failed to attend to avoid the stigma associated with going to school with their younger peers. There were no adult education programmes or accelerated learning programmes provided in the camps to help children catch-up in school therefore many children who lost school time during the war (including children who were abducted) were forced to (re) join with classmates who were much younger than them. Due to their older age, children felt out of place in school and many of them subsequently dropped-out. Many parents (60% of individual interviews with adult women) indicated that girls being out of school coupled with the fact that children were no longer accompanying and assisting their mothers in the garden, increased girls’ vulnerability to predation during the day.

#### Vulnerability at night

The night-commuting phenomenon during the war, where thousands of children flocked from their villages to Gulu town to sleep in churches, hospitals, and on verandas in order to avoid abduction and other violence, is well documented [26]. At its peak in the spring of 2004, there were 40,000 children commuting every night. Children would walk several kilometres to town every night to sleep; in the morning they would walk back home, go to school, and then come back into town to sleep again. Various Non-Government Organizations (NGOs) responded to the crisis by organizing sleeping shelters in urban centres. At that time, NGO reports highlighted concerns about the predation and sexual abuse/violence of girls as they moved unescorted from villages to the sleeping centres [26]. Through our interviews, we uncovered another concerning ‘night-commuting’ trend that was characteristic of the IDP camp situation and was of great concern. Girls and boys were moving away from their families’ huts at dusk to sleep in other huts within camp perimeters. For the most part, many children were moving to circumstances where an older cousin was the only person providing supervision to a hut full of children, both girls and boys. The explanation for this ‘internal’ night-commuting by children in IDP camps is two-fold. First, when children were old enough to understand their parents’ need for sexual privacy they traditionally did not sleep in their parents’ hut but rather in their grandmothers’ hut or the bachelors’ hut [24]. It appears that children were still following these cultural traditions in IDP camp settings. Second, if families were living on the periphery of the camp, their children were at increased risk for abduction by the very nature of the location of their hut. For security reasons, parents would attempt to negotiate a safe hut for their child to sleep in closer to the center of the camp. Some families, early in camp settlement, may have secured enough land to build two huts for their own families. Frequently, these huts would come up for ‘rent’ to those families who needed alternative arrangements for their children. According to both the women and girls we interviewed it was during this ‘internal’ night-commuting when girls were most vulnerable to predation by men who would be offering food and clothing in exchange for sex; many girls we interviewed (52% of individual interviews with adolescent girls) reported that this is when their sexual debut occurred. The following quotes illustrate this concerning trend:

“If you are old, you cannot share room with your parents; you will go and sleep with your relatives, so you are showing them respect.” [(I) Adolescent Girl]

“Their lives are spoilt. At night they roam about the camps, going out to visit their boy friends, where they spend the night and come back in the morning. This is because they don’t sleep in the same house with their parents. So the parents don’t know what the girls do at night.” [(I) Adult Woman]

### THEME III: Access to reproductive and sexual health information/services

Various sources of sex/HIV/AIDS information in the IDP camps were identified by discussants. Participants reported receiving information from: parents; elder sisters and brothers; teachers; (sex) magazines; Straight Talk (a monthly article in the New Vision newspaper); radio programmes; health workers; sexual partners; peers; church, and; pornographic video shows. However, a common finding was that many of these information sources were not necessarily cognizant of the current realties of adolescent girls living in displacement camps, which in-turn diminished their overall benefit. As one girl explains:

“When they [family planning services] are teaching about condoms, they usually restrict it to people of 18 years and above. They are the ones who are advised to use it. The use of family planning is for married women (those with husbands) not for girls…young girls in the ages of 12–14 years don’t have any knowledge about condoms.” [(G) Adolescent Girl]

Similarly, FM radios were reported to be sources of sex/HIV/AIDS information; they communicated information on how to use condoms, avoid STIs/HIV/AIDS and unwanted pregnancies, and encouraged blood testing for HIV. However, some adolescents noted the shortcomings of these campaigns including redundant repetition and incongruity between the assumptions of the broadcasters and the children’s realities. For instance, radio presenters took for granted that condoms and medical services were readily available in camps, which was rarely the case. In fact, an over-whelming majority of adolescent girls (91% of individual interviews) found it difficult to obtain condoms in the camps. Furthermore, most adolescents lived in homes that could not afford to buy radio batteries, leaving many children without access to the radio programmes.

Churches were also identified as sources of sex-related information for young people. However, research participants argued that churches only reached those that were preparing for marriage and that very few adolescents organized their sexual relationships and marriages through church anymore. Apart from preparation for marriage, churches were noted to lack adolescent-specific sex education programmes because the church never perceived adolescents as sexually active before marriage. This, however, was no longer the case for many displaced young people.

While some literature on sex had found its way into the camps, most discussed sexual performance but not sexual education. Most magazines emphasized sex styles, methods of luring women and men into sex and other issues related to sexual performance, not anticipation of and prevention of potential risks. Further, although Straight Talk articles found in newspapers available in the camps contained useful sexual information, access to this information required purchasing the newspaper, an impractical and expensive luxury for most adolescents. Moreover, the articles were written in English making it difficult especially for those adolescents who were out of school to benefit from their content.

Although many participants (68% of individual interviews) reported access to information on HIV/AIDS, very few (6% of individual interviews) noted sufficient access to information on puberty, sexuality, condoms, abortion and pregnancy. Additionally, the majority of participants (73% of individual interviews) articulated an urgent need for sexual health information and services that were adaptive to the current needs of adolescent girls and could support girls in translating their knowledge and awareness of HIV/AIDS into prevention behaviour. As one woman explains:

“Other sex related information in this area is urgently needed so that our youths are helped to bring changes in their lives…but without that then at 12 years, 13 years you find all girls pregnant.” [(G) Adult Women]

## Discussion

It is well known that displaced people, particularly girls and women, are at a much higher risk than the rest of the population for communicable diseases and other health concerns including HIV/AIDS [27]. However, the combination of economic, social, biological, and behavioural factors that increase adolescent girls’ sexual vulnerability in times of war and render them disproportionately susceptible to HIV/AIDS is not yet perfectly understood.

In study interviews many women commented that, “we have lost control of our children” and “we are experiencing complete familial and cultural breakdown”. This research process has furthered our understanding of parental and extended family frustration and anguish regarding the vulnerability of their daughters living in squalor, impoverished and hungry. The lack of control they feel they have over the well being of the girl-child stands in stark contrast to the systems of protection in existence prior to IDP camp relocation. In many ways women – grandmothers, aunties and mothers – were the active witnesses/player/consenters in the transition of their daughters from adolescence to womanhood. However, the personal security and support offered to girls by their families was virtually extinguished upon the advent of war. Traditional mentoring systems and cultural norms that previously governed girls’ sexual behaviour and provided cultural cohesion and guidance in supporting and protecting young girls from risky behaviours, have largely been eroded by war-induced displacement. In IDP camps the mentoring systems are limited by the close proximity between families, thus restricting the traditional comfort that had been associated with the grandmother’s hut. Further, cultural norms governing girls’ sexual behaviour have disintegrated because the family unit is now broken-up and dispersed among other families. These findings demonstrate that children no longer have a culturally ‘safe’ place to go for information on sexual matters.

Research suggests that the erosion of belief systems and a shift in cultural norms governing girls’ sexual behaviour in times of conflict helps to explain their heightened sexual vulnerability during war [28]. This breakdown increases the likelihood of early sexual debut, unprotected sexual activity and larger numbers of sexual partners [17,29]. It is clear that the erosion of family and other social and cultural processes needs to be meaningfully addressed through appropriate programming if the vulnerability of girls in conflict settings is to be mitigated. The Government of Uganda is reinstating plans for ‘resettlement’ in the northern region as relative peace prevails [30]. Post-conflict prevention planners must recognize that the realities of girls will not necessarily change when peace ensues. Although the long-term effects of conflict on children is rarely discussed, available evidence suggests that girls’ vulnerability to sexual and intimate partner violence does not necessarily end with the cessation of armed conflict; in fact in many instances their vulnerability is exacerbated by reconstruction programmes that fail to specifically target their needs [16,17]. Therefore, any planning for resettlement must incorporate the adequate provision of basic humanitarian services, including HIV prevention programming, particularly for children already traumatized by war. Moreover, as people are leaving camps and moving back to their home villages there is more room to build multiple huts for one family. Hence, families should be encouraged and supported in building several huts for their homestead and resettling according to clan and/or in close proximity to members of extended families (i.e. cluster homes), as the Acholi once resided before the war. Living with and/or near family members will help reinstate traditional child protection components of the Acholi compound, family and moral value systems while also providing increased security for adolescent girls. Indeed, research suggests that in places where values and principles mirrored by familial and clan obligation remain strong despite the war, kinship-related interventions may provide the only opportunity for consistent programming in conflict settings [31].

Devoid of any means of livelihood in the camps, a people of agriculturalists and livestock breeders have been reduced to near total dependence on donated food and other humanitarian aid. The anguish of poverty and the extreme difficulties women and girls face to provide for their families in IDP camps is demonstrated in our results. With no land to cultivate or crops to harvest the opportunities are restricted for girls who are out of school to make money to procure items that they consider important (including soap, Vaseline, panties and dresses). These results are worrisome because of the potential for girls participating in transactional or survival sex relationships and early marriages, which inherently increase adolescent girls’ sexual vulnerability [32]. Mothers in this study indicted selling portions of WFP food rations to buy the things they felt their daughters needed to prevent the initiation of sex for exchange relationships. It is clear that in order to alleviate the vulnerabilities of adolescent girls in times of conflict, women and girls must be supported in providing for their families. The provision of marketable skills training in camp settings is therefore very important. However, appropriate skills training relevant to the market economy and camp living must be considered. For example, training in tailoring might not be relevant because there is no one to buy new clothing; however, with a micro-credit loan girls might be able to buy a bail of used clothing and sell it in a market. By decreasing the dependence of girls on men for economic security, adolescent girls’ sexual vulnerability will intrinsically be alleviated [17,32]. Therefore, local and international NGOs must be encouraged to initiate and support appropriate marketable skills training (e.g., hairdressing and catering courses) to female camp residents, particularly adolescent girls who are not in school.

According to the Uganda AIDS Commission and reinforced by our study results, Ugandan youth begin sexual activity at young ages and with little information on sexuality thereby increasing their risk of contracting HIV/AIDS [33]. Traditionally, premenstrual sex was forbidden in Acholi culture and age of sexual debut ranged from 16–18 years. The diminishing age of sexual debut for girls observed in this study, reported by many women to be between 12–14 years old, is telling of adolescent girls’ increased sexual vulnerability in times of conflict. Often out of economic need girls are having sex earlier and with older men, and some men seek younger girls as sexual partners in the belief that they are more likely to be HIV-negative. Age disparities in sexual relationships increase the likelihood of sexual coercion and inhibit girls’ ability to control the terms of their sex lives including negotiating condom use, subsequently increasing their exposure to HIV [34].

Furthermore, literature suggests that early sex may increase the likelihood of early marriage and pregnancy, signalling the end of education for most girls and leaving adolescent girls even more at risk of poverty and sexual exploitation [35]. Our study results corroborated this literature, as the primary reasons reported by girls for dropping out of school were marriage and/or pregnancy, in addition to lack of money for school-related supplies (uniforms, books, meals). Moreover, our results demonstrated that girls who were not in school were being left behind in the camps for most of the day instead of accompanying and assisting their mothers in the gardens, due to fear of abduction. These circumstances left out-of-school girls unsupervised in camps during the day increasing the potential for sexual predation by men offering food and clothing in exchange for sex. A report by UNICEF found that after families, schools are the next perimeter of a protective environment for children [36]. Schools can be a powerful protective force in most children’s lives, especially for girls, as schools physically remove children from potential harm for much of the day and help children learn skills and gather information to protect themselves and delay on-set of sexual activity. The longer girls are kept in school their vulnerability to HIV infection lowers by approximately thirty per cent [36]. Consequently, in-light of the widespread benefits of education, including the physical protection and sexual security that schools can provide to adolescents, every effort must be made to ensure displaced girls have consistent and stable access and support in their academics in times of conflict. Therefore, educational responses from the Ugandan Government and NGOs in northern Uganda must prioritize the provision of policies and programmes that support adolescent girls in attending and maintaining their in-school status, particularly young mothers with children and girls who are married. Currently, due to restrictive national policy, girls who get married or become pregnant are generally forced out of school. After substantial time away, the majority of these girls do not end up re-entering the school system, mainly due to the stigma associated with older girls re-entering school with their younger peers and, lack of childcare support for young mothers [37]. As such, it is imperative that the Ministry of Education in Uganda restructure current educational policy so that young girls who become pregnant or get married are not forced to leave school. Moreover, this policy change must coincide with the development of special bridging centres (attached to government schools) for young mothers where they can continue with their formal education while their children are cared for. In addition, the Ministry must ensure that older girls who drop out of school are afforded the opportunity of accessing accelerated learning programmes, complementary educational initiatives which allow young people to catch-up with their studies relative to their peers, in-turn increasing the chances of school leavers re-entering the formal education system. Finally, the Government of Uganda and the Ugandan Ministry of Education must work with the districts and sub-districts in the North, as well as local NGOs and international donor agencies, to provide funding for all the ‘extras’ that keep children from entering/re-entering school or force school-going children to subsequently drop-out (i.e. uniforms, books, meals).

Our study results demonstrated that girls were also vulnerable at night; our analysis uncovered a very concerning ‘internal’ night-commuting phenomenon whereby, due to privacy and security concerns, children moved from their family hut at night from the outskirts of camps to sleep in more central locations within their camp’s perimeters in a friend’s or relative’s hut without any adult supervision. Many parents reported the increased vulnerability of their daughters, in particular, to sexual predation during this internal nighttime movement. Unfortunately, the hundreds of children moving at night within the perimeters of the camps has not been highly recognized as a night-commuting phenomenon in and of itself, and has therefore not been properly assessed from either a research or a policy perspective. A concerted effort by researchers, policy makers and, programme planners to address these unique circumstances is urgently required.

In Northern Uganda, the Information, Education, and Communication (IEC) campaigns focused on the dissemination of HIV/AIDS information can be considered successful, as the majority of our study participants had a high awareness of HIV/AIDS and a high knowledge level of how to prevent infection. Yet what is apparent in our analysis is that girls are having difficulty translating knowledge of how to prevent infection into risk minimization practice. Many girls reported that condom availability in the camps was inconsistent or unavailable altogether. Moreover, several participants expressed the need for sexual health information and services in camps that was cognizant of the current realities and needs of adolescent girls and that would support them in the translation of HIV/AIDS awareness and knowledge to personal modification of sexual lifestyles. Combined, these findings are an indicator of the lack of appropriate sexual health programming that exists in camps and are of concern. With limited access to knowledge and means to protect one’s self, girls are inherently vulnerable to high-risk sexual activity [38]. It is well documented that war-related insecurity has diminished opportunities for NGOs and other organizations providing prevention programming to have a strong and consistent presence in displacement camps [17,39,40]. However, research demonstrates that reduced access to reproductive and sexual health services including relevant sexual health information increases the vulnerability of adolescents in particular [29]. Prevention programming planners in camps must realize that early sex, early marriage, and early pregnancies are the realities of adolescent girls living in displacement camps. Appropriate responses and innovative community-driven solutions acknowledging this actuality are urgently required to ensure adolescent girls have support and access to the information and means they need to protect themselves. For example, Spittal et al. (2008) developed the *Wayo* Programme, a reproductive health initiative in northern Uganda that trained women from the community to assume traditional Acholi, *wayo*-like counselling roles for the purposes of passing on sexual education and HIV prevention information from adults to younger women [31]. This is a highly successful and sustainable initiative because the women themselves are chosen by the community to become trained in STI prevention, condom use and reproductive health issues. They essentially resurrect roles that carried traditional respect; roles, which have now been lost or eroded due to prolonged conflict and displacement. Similar interventions that are built solidly upon indigenous knowledge and supported by the community must be supported and expanded. Furthermore, consultations must occur between the *Wayo* Programme, girls at risk, and other NGO and service providers in northern Uganda, including Save the Children, The AIDS Support Organization (TASO) and Straight Talk, on ways to increase access to condoms, condom information, VCT, STI treatment and antiviral care in camp settings. Strengthening reproductive health service provision to adolescent girls in times of conflict would improve their health outcomes considerably [17].

## Conclusions

Departing from the recognition of a paucity of knowledge on the distinct vulnerabilities of adolescent girls in times of conflict, this study sought to explore the sexual vulnerabilities of displaced adolescent girls in Northern Uganda. Our analysis identified the erosion of traditional Acholi mentoring and belief systems that had previously served to protect the girl child’s sexuality, in addition to a collapse of livelihood in the face of war. These factors coupled with: being left in camps unsupervised and not in school during the day; movement within the perimeters of the camp at night away from the family hut to sleep in huts closer to the center of the camp due to fear of abduction and cultural considerations pertaining to privacy, and; inadequate access to relevant and adaptive sexual health information and programming in the IDP camps, have all contributed to adolescent girls’ heightened sexual vulnerability and subsequent enhanced risk for acquiring HIV/AIDS in conflict settings. It is safe to say that the lives of Acholi girls and women have been irrevocably impacted by over two decades of war and displacement. Conflict prevention planners, resettlement programme developers and policy-makers must recognize adolescent girls affected by armed conflict as having distinctive needs, which may not be the same as women, children or adolescent boys. Moreover, the unique needs of conflict-affected adolescent girls require distinctive responses.

## Competing interests

The authors declare that they have no competing interests.

## Authors’ contributions

PMS and NKS developed the study design with assistance from HM, GO and SA; SA collected the data with oversight from NKS, HM and GO; SHP preformed the data analysis and drafted the manuscript; SHP, PMS, NKS, HM and SA were responsible for the interpretation of results and final revisions to the manuscript. All authors read and approved the final manuscript.

## Pre-publication history

The pre-publication history for this paper can be accessed here:

http://www.biomedcentral.com/1472-698X/12/38/prepub
